# Effect of vineyard soil variability on chlorophyll fluorescence, yield and quality of table grape as influenced by soil moisture, grown under double cropping system in protected condition

**DOI:** 10.7717/peerj.5592

**Published:** 2018-09-04

**Authors:** Sangeeta Mitra, Muhammad Irshad, Biswojit Debnath, Xiaocao Lu, Min Li, Chandra Kanta Dash, Hafiz Muhammad Rizwan, Zhipeng Qiu, Dongliang Qiu

**Affiliations:** 1 College of Horticulture, Fujian Agriculture and Forestry University, Fuzhou, Fujian, China; 2 College of Plant Protection, Fujian Agriculture and Forestry University, Fuzhou, Fujian, China; 3 Xiamen Lunong Agricultural Science and Technology Co., Ltd, Xiamen, Fujian, China

**Keywords:** Summer black, Grapevine, Anthocyanin, Flavonoid, Antioxidant, Phenolics

## Abstract

Environmental factors greatly influence grape quality. Among them, the effect of within-vineyard variability of soil in relation to soil moisture on table grape under protected condition has rarely been studied. In this present research, we investigated the influence of soil variability, in relation with soil moisture on chlorophyll fluorescence, yield and quality attributes of the “Summer Black” (*Vitis vinifera* L. × *V. labruscana* L.) table grape, popularly grown under double cropping system in protected covering in the southern part of China. The vineyard was divided vertically into three sites (lower, middle and upper, 192, 202 and 212 m above sea level, respectively) and data on soil moisture and other yield and quality parameters were recorded. Among the three vineyard sites, middle site resulted in higher yield compared to the upper and lower sites during winter and summer cropping cycles. However, compared to regular summer cycle, winter cycle provided grapevines with higher quality attributes. Polyphasic OJIP fluorescence transient exhibited a considerable increase in fluorescence intensity at J, I and P phase in the upper and middle sites compared to the lower site due to variation in soil moisture in both seasons. Values of fluorescence parameters including minimal fluorescence, relative variable fluorescence at phase J and I, the maximal quantum yield of photosystem II were also influenced by soil moisture in different sites. Different sites also exhibited a significant difference in total phenolics, flavonoid, antioxidant activity and individual anthocyanin which was influenced by available soil moisture. The present study shows that chlorophyll fluorescence OJIP transient can be used as a sensitive indicator to determine the moisture stress in grape grown in a varied soil. Double cropping proved to be a powerful technique to improve the fruit quality. This result may be useful for the table grape growers to better utilize the vineyard soil variability with water management to get higher yield and quality table grape under protected condition.

## Introduction

Grapevine (*Vitis vinifera* L.) is an important perennial fruit crop that grown worldwide. While almost 50% of grapes are used to make wine, one-third is consumed as fresh fruit (36.91%) and the rest is dried (8.5%), consumed as grape juice (5.1%) or stored in the form of grape musts (whether concentrated or not) (FOCUS, 2016). After citrus, bananas and apples, table grape rank fourth which is consumed as a fresh fruit with an estimated production of 26.8 million tonnes, and China ranks the largest table grape producing country in the world accounting for 34% of the global table grape production (FOCUS, 2016). Grapes are rich in antioxidants having polyphenolic compounds like anthocyanin, resveratrol and phenolics which are known to play an important role scavenging free radicals (Sarma & Sharma, 1999) and have a protective role against cardiovascular diseases, prostate and colon cancer (Merten-stalcott et al., 2008), cold temperature and fungal/viral infections (Williams, Grimes & Phene, 2010).

Table grape quality depends upon both biophysical and biochemical traits like size, color, firmness, TSS (total soluble solids) and acidity (Champa, 2015; Williams, Grimes & Phene, 2010). Various environmental factors such as climate, soil type, altitude, topography, water availability and temperature have been reported to influence grape and wine quality substantially (Bramley, Ouzman & Boss, 2011; Gomez-Miguez et al., 2007; Koundouras et al., 2006; Vilanova et al., 2012). For example, altitude has been observed to affect the mesoclimate thereby influencing the grape maturation (Mateus, Machado & Freitas, 2002), wine sensory profile (Alessandrini et al., 2016) and flavonoid and polyphenolic composition (Xing et al., 2015). Jiang et al. (2013) compared the aroma composition of two wine grapes, Merlot wine and Cabernet Sauvignon from four different regions of different altitudes in China and found a significant difference in the compounds analyzed from the different sites. Unlike high altitude, which has been found to have strong influence on grape quality (Alessandrini et al., 2016), soil variability within a vineyard even with lower elevation may also influence grape yield and quality which has not been studied previously.

Other than genotype, environmental condition and cultural practices, the majority of studies on table grape indicated that grape quality parameters are also influenced by irrigation (Permanhani et al. (2016) and references therein). Changes in water availability have been recorded to change grapevine physiology, which in turn, can affect yield and quality (Esteban, Villanueva & Lissarrague, 1999). Cultivation of table grape is characterized by high water productivity as well as intensive use of water (Permanhani et al., 2016). In comparison to wine grapes, table grape annual production is also very high (17.1–49.9 ton ha^−1^) which depends on very high inputs of water (Molden et al., 2010; Zúñiga-Espinoza et al., 2015). Water stress has an adverse effect on plant growth, metabolism and yield which can decrease crop productivity (Chaves, Flexas & Pinheiro, 2009; Lawlor, 2002; Wang, Vinocur & Altman, 2003). Water stress may irreversibly reduce photochemical efficiency by damaging the photosynthetic apparatus (Zulini et al., 2007). The chlorophyll a fluorescence OJIP transient is an efficient technique for studying different physiological characteristics of structure and activities of the PSII (Strasser, Tsimilli-Michael & Srivastava, 2004), which has been extensively used to show the changes in the photosynthetic system of plants caused by any environmental stresses (Guo & Tan, 2015; Maxwell & Johnson, 2000; Zushi, Kajiwara & Matsuzoe, 2012). OJIP transient represented by the O, J, I and P steps, in relation to the redox state of the PSII primary acceptor (Strasser, Srivastava & Tsimilli-Michael, 2000; Strasser, Tsimilli-Michael & Srivastava, 2004). Besides, OJIP transient is easy, simple, rapid and non-destructive testing method for chlorophyll-containing sample (Strasser, Srivastava & Tsimilli-Michael, 2000; Strasser, Tsimilli-Michael & Srivastava, 2004). Many authors reported chlorophyll a fluorescence as a reliable technique to monitor physiological changes in the plant and used as a stress indicator (Kalaji et al., 2012; Oukarroum et al., 2007). Previous studies reported a decrease in the potential quantum efficiency of PSII (F_v_/F_m_) indicating the photoinhibitory damage due to the effect of water stress (Epron, Dreyer & Bréda, 1992). OJIP transient, a powerful technique for measuring water stress on photosystem II (PSII) has never been tested against vineyard with variability in soil which may affect the grape production and quality. Grape is mainly a summer crop which undergo dormancy from fall to spring after that singly pruned and harvested in the summer season. In Southern China, which is characterized by high temperature and heavy rainfall during grape veraison to harvesting time which ultimately reduce the yield and quality of grapes with increased prevalence of fungal diseases (Bai et al., 2008). In table grape production, protected cultivation in a vineyard in different regions and climates is nowadays a commonly used practice with higher water use efficiency and better berry quality (Novello & De Palma, 2008; Roberto, Colombo & De Assis, 2011). Because covering with plastic film protects vines and fruits from an adverse condition like wind, rain, hail, frost, scorching sunlight, pest and diseases (Du et al., 2015; Genta et al., 2010; Novello & De Palma, 2008). Chavarria et al. (2009) studied the influence of plastic cover on yield components of grape cultivar *Moscato Giallo* and concluded that plastic cover promoted yield and production stability without affecting the pulp/skin ratio. In a study, Rana et al. (2004) compared uncovered vine with plastic film and thin net as a covering strategy in table grape vines (cv. “Italia”) to determine the effects of covering on water requirements (evapotranspiration, etc.). They found high evapotranspiration in uncovered vineyard compared to vineyard covered by plastic. In the uncovered vineyard, evapotranspiration was 6.4 mm day^−1^ after irrigation and decreased rapidly to about 4 mm day^−1^ just after 9 days whereas, in case of vineyard-covered by plastic, evapotranspiration was 3 mm day^−1^ after irrigation and it was decreased very slowly before it reached to 1.9 mm day^−1^ after 24 days of irrigation. In South China, another popular viticulture is practiced which is known as “Double cropping viticulture system” where compound buds are forced to break dormancy with pruning and application of hydrogen cyanamide in early February which help to harvest grape in June–July as a summer season crop (Bai et al., 2008; Chen et al., 2017; Lin et al., 1985). After the harvest, again the vines are pruned and chemical applied in August to get second crop in December–January as a winter season crop. Two crops in a year was achieved successfully by Taiwan with the help of defoliation, pruning and chemical treatment (Lin et al., 1985). Recently in Southeast Brazil, double-pruning proved to be a reliable technique to produce good quality wine grapes with higher yield, sugar accumulation and anthocyanin from winter harvest compared to regular summer harvest (Favero et al., 2011).

In this current study, we investigated the effects of within-vineyard soil variability on chlorophyll fluorescence, yield and quality parameters of table grape grown under double cropping system a unique viticulture practice where grape is harvested twice, during winter and summer. To better understand the effects of vineyard soil variability we focused on soil moisture associated with different sites by keeping the other agronomical and environmental factors as uniform as possible. Specifically, we examined: (1) whether different sites within-vineyard affects yield and quality of grape in relation to soil moisture; (2) the behavior of PSII using chlorophyll fluorescence JIP test to determine the water stress for the grapevine with soil variability; and (3) how seasonal variability interact with soil moisture to affect table grape production and quality traits.

## Materials and Methods

### Experimental site, design and double cropping viticulture practices

**Figure 1:**
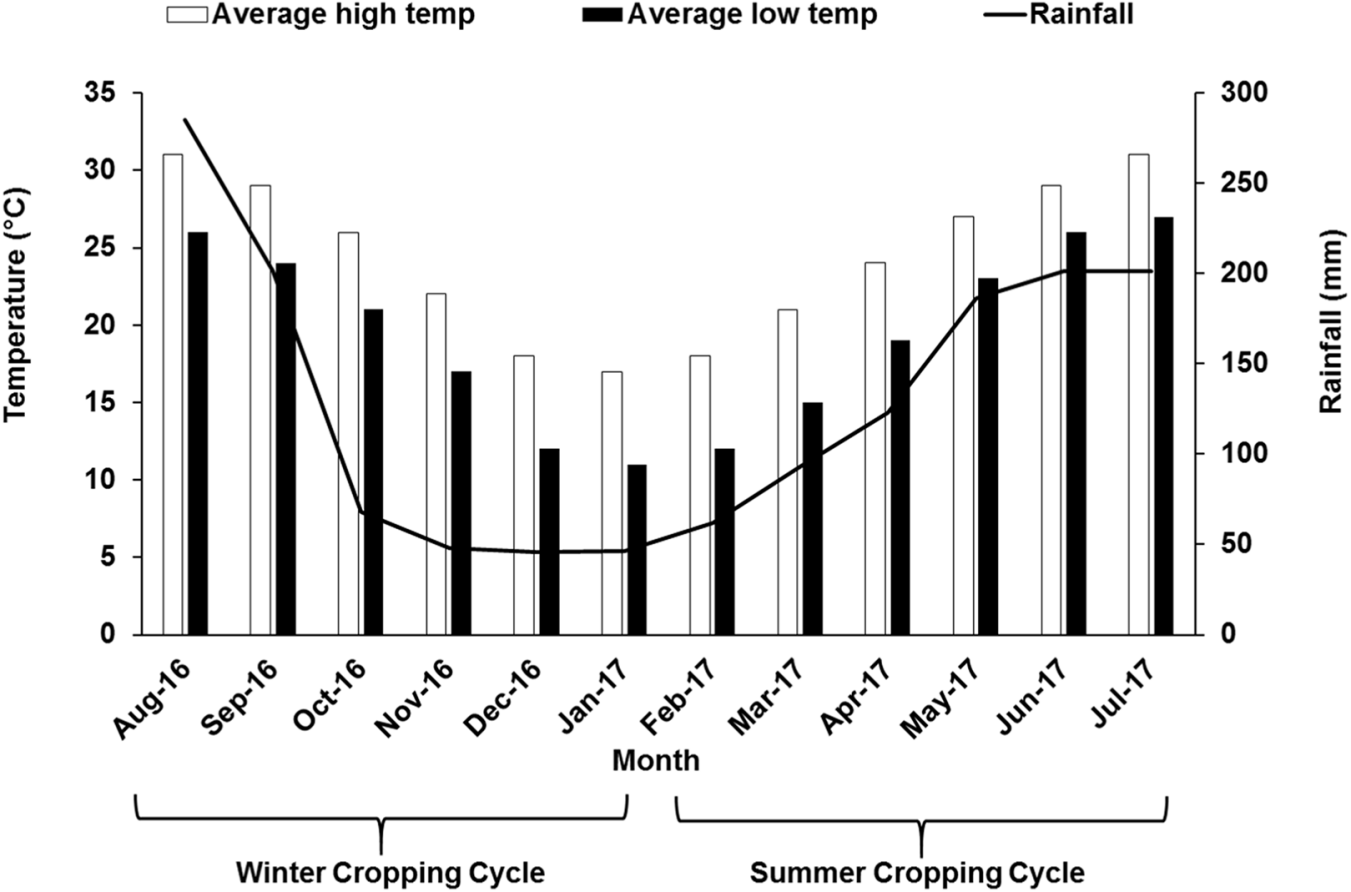
Monthly air temperature and rainfall of the experimental site during winter and summer cropping cycle.

The study was performed during the 2016–2017 growing season in a 7-year-old commercial vineyard of “Summer Black” (*V. vinifera* L. × *V. labruscana* L.), owned by Fujian Jiujiu Tianchen Eco-Agri LTD located in the county of Anxi (Fujian, China) (25°00′N, 117°59′E) usually characterized by humid monsoon with abundant sunshine. The formal approval was obtained before the experiments were conducted in the field. In south China, grape cultivation is mainly dominated by double cropping system, where the vines are pruned two times and grapes are harvested twice within a year. At first, to get crop in the summer season cycle, grapevine was pruned and forced with 2.5–3.0% hydrogen cyanamide in early February when the temperature was above 10 °C. Around late March flowering was initiated, berry formation and veraison were around late-April and mid-May, respectively. Summer crop was harvested in June–July. After that, the vines were again pruned and forced in early-August, which flowered in September, followed by the berry formation and veraison stage during October and November leading to harvest in December–January. Different growth stages during two different seasons are illustrated in Table 1. The vineyard is established on a sloppy land. Vines in the vineyard were maintained under a tunnel covered with plastic polyethylene film to act a shelter on top. Water was being supplied through drip irrigation system with drip emitters at a rate of 4 L m^−2^ h^−1^ prior to bud break and fruit setting stage. No irrigation was supplied after veraison up to harvest. In this study, the vineyard was divided vertically into three sites at different height: the first site was selected at 192 m above sea level (“lower” site), the second site was around 10 m above first site (“middle” site) and the third site was at around 20 m above the first site (“upper” site). The vineyard soil is almost similar in characters and classified as sandy loam. Each site (upper, middle and lower) was considered as one treatment and each site was divided into three blocks with 10 vines from each block, that is, in total 30 plants were selected from each of the upper, middle and lower sites for data collection. Areal temperature and rainfall pattern data for the study year have been illustrated in Fig. 1.

**Table 1:** Growth stages of “Summer Black” table grape grown in double cropping viticulture system.

Season	Bud break	Flowering	Berry formation	Veraison	Harvest
**Summer**	Mid-February	Late-March	Late-April	Mid-May	June–July
**Winter**	Mid-August	September	October	November	December–January

### Determination of soil moisture

Soil moisture was determined by Gravimetric methods. The soil samples were collected from 0 to 20 cm and soil from 20 to 40 cm depth with the help of a soil auger at 15 days after veraison. Each sample was a composite of two sub-samples taken from both sides of vine at 0.30 m far from emitters. It is reported that much of the grapevine root system represents the soil volume covers 0.4 m depth (Bassoi et al., 2003), hence, soil collecting depths were chosen to represent the biologically active top soils and the subsoil containing most of the grapevine roots. Soils were collected from the field with airtight aluminum containers and taken to the laboratory. The soil samples were weighed and then dried in an oven at 65 °C until all the moisture was driven off. After being removing from the oven, they were cooled down slowly to room temperature and weighed again. The difference between initial and final weight is the amount of moisture in the soil. The moisture content in the soil was calculated by the following formula: moisture content (%) = ((initial weight−oven dry weight)/oven-dry weight)) × 100.

### Measurement of leaf OJIP transients

The transients were measured on fully expanded grapevine leaves by a portable plant efficiency analyzer, Handy-Pea (The Hansatech Instruments Ltd, Norfolk, UK) at 15 days after veraison. Randomly nine leaves from each replicate were selected for measurements of chlorophyll a fluorescence. Before the measurements, all the leaves were dark adapted for 3 h at room temperature. The OJIP transient was induced by the red light with maximum intensity of >3,000 μmol m^−2^ s^−1^ at leaf surface provided by an array of three light-emitting diodes at peak wavelength 650 nm, spectral-line half width 22 nm in a time scan from 20 μs to 1 s after the onset of irradiation with a data acquisition rate of 100 readings with a record length of 1 ms–300 s. Measured OJIP transients were analyzed according to the JIP-test equations (Smit et al., 2009; Strasser, Tsimilli-Michael & Srivastava, 2004). From the original measurements the following values were used: the minimal fluorescence intensity at 20 μs when all PSII reaction centers (RCs) are open (the O step, considered as F_o_); the intensity at 300 μs which is used for calculation of the initial slope (M_o_), defined as the net ratio of RCs closure; the intensity at 2 ms (the J step, F_J_); the intensity at 30 ms (the I step, F_I_), and the maximal fluorescence intensity when all PSII RCs are closed (the P step considered as F_m_).

### Berry sampling and fruit analysis

To determine the optimum harvesting time, grapes from different experimental plot were periodically tested with a digital hand refractometer and harvesting time was considered when the TSS content of berries attained approximately 16 °Brix. Physicochemical analysis of the grapes was accomplished by collecting 10 clusters from each replication of a treatment, and 12 berries were collected from each cluster (four from each upper, middle and lower portion) in total 120 berries per replication. Vine yield per tree (kg) was expressed as the multiplication of the total number of cluster per tree and the total weight of the cluster (g).

The collected 120 samples were divided into two parts. The first 60 berries were again divided into three biological replicates of 20 berries each, which were then used to measure the physical characters of the berries by recording diameter of berries, berry length and individual berry weight and after that the berries were crushed into juice for measuring the TSS, pH and Titratable acidity (TA). For harvesting berry, increased concentration of total soluble solid is a reliable indicator (Coombe & McCarthy, 2010). TSS was measured using a digital refractometer (DR301-95, Krüss Optronic, Hamburg,Germany) and the result was expressed in °Brix. pH was measured by pH meter. TA was measured by the potentiometric method, where the prepared juice was being titrated with 0.1 N NaOH solutions until the pH value reached at 8.2 on the pH meter. The results were calculated in percentage of tartaric acid. Skin from the rest 60 berries were manually peeled and frozen immediately in liquid N_2_ and kept at −80 °C until further analysis.

### Extraction and estimation of total phenolics, flavonoid and antioxidant activity

The berry skin samples at first were ground in liquid N_2_ with the help of a mortar and pestle, from that powdery sample 0.5 g was homogenized in 10 mL of 80% chilled ethanol. The extracts were centrifuged at 12,000 rpm at 4 °C for 20 min. Then the supernatant was filtered on a filter paper, collected and used for the estimation of total phenolics, flavonoid and total antioxidant capacity.

Total phenolics content in the sample was determined using the Folin–Ciocalteu colorimetric method according to Huang et al. (2009) with minor modification. Briefly, 0.5 mL of aliquots and standard Gallic acid of different concentrations (20, 40, 60, 80, 100 μg/mL) was added into a 10 mL tube in which 4 mL of distilled water and 0.5 mL of Folin–Ciocalteu’s reagent (FCR; Sigma Chemical, St. Louis, MO, USA) was added previously. The tube was shaken. About 5 min later, one mL of 7% sodium carbonate (Na_2_CO_3_) was added to the mixture. The mixture was kept in dark condition at room temperature (25 °C) for 120 min. After that, an intense blue color was developed. The absorbance was then measured against a blank at 765 nm using UV–VIS spectrophotometer (TU-1810; Beijing Beifen-Ruili Analytical Instrument (Group) Co., Ltd., China). The measurement was performed in triplicates. The data for total phenolics contents were expressed milligram GAE 100 g^−1^ berry skin fresh weight (FW) (Gallic acid equivalent).

Total flavonoid content was measured by a colorimetric assay according to Huang et al. (2009). A 0.5 mL of aliquots and 0.5 mL standard quercetin solution (100, 200, 400, 600, 800, 1,000 μg/mL) was added into a 10 mL tube containing four mL of water and 0.3 mL of 5% NaNO_2_ was added into it, then the mixture was allowed to stand for 5 min at room temperature. After that, 0.3 mL of 10% AlCl_3_ was added to the mixture. After 5 min, two mL of 1M NaOH was added. Distilled water was added to make up the total volume up to 10 mL and mixed well. Orange yellowish color was developed. The absorbance was measured spectrophotometrically at 510 nm against a blank. All the procedures were performed in triplicate. The calibration curve was made using standard quercetin. The result of total flavonoid was expressed as milligrams quercetin equivalents (QEs) per 100 g berry skin FW (mg QE/100 g of FW).

The total antioxidant capacity was measured by the Ferric Reducing Antioxidant Power (FRAP) assay according to Benzie & Strain (1996). FRAP reagent includes acetate buffer (30 mM, pH 3.6), TPTZ (10 mM in HCl 40 mM) and FeCl_3_·6H_2_O (20 mM). The FRAP reagent solution was prepared by adding acetate buffer, FeCl_3_·6H_2_O and TPTZ using 10:1:1 (v/v/v) ratio. We mixed 30 μL of Skin extracts with 2,900 μL of the FRAP solution and 70 μL Mili-Q water then allowed to stand for 30 min in the dark condition at room temperature (25 °C). After that, the absorbance of the colored solution was determined at 593 nm against a blank. The standard curve was prepared by using one mM Trolox and result was expressed as millimolar TE/g berry skin FW.

### Extraction and estimation of total and individual anthocyanin

Anthocyanin extractions were performed with a protocol similar to that of Liang et al. (2011) with slight modifications. Briefly, the frozen berry skin samples were ground in liquid nitrogen with a mortar and pestle. From there, a 0.5 g powdery sample was homogenized in 10 mL of 1% HCl-methanol solution and further mixed. It was vortex for 1 min and then kept in a shaker at 250 rpm for 2 h at 4 °C in the dark. Then, centrifuged at 4 °C for 20 min at 12,000 rpm. Then the supernatant was collected and kept at −40 °C until the time of analysis. This extract was used for the estimation of total and individual anthocyanin.

The total anthocyanin content was measured by pH differential method using a protocol similar to that of Giusti & Wrolstad (2001). The total required amount of skin extract was added into two tubes, and these extracts were diluted 10× with KCl (0.025M) buffer at pH 1.0 and CH_3_CO_2_Na·3H_2_O (0.4M) buffer at pH 4.5. The absorbance was measured at 520 and 700 nm against distilled water as a blank, respectively, and calculated using the equation A = (A_520_–A_700_) pH1.0 − (A_520_–A_700_) pH 4.5. Each anthocyanin extract was diluted to get the sample in the buffer at pH 1.0 with an absorbance <1. The resultant anthocyanin content was expressed as milligrams of malvidin-3-monoglucoside equivalence per killogram of berry FW.

The chromatographic analysis of individual anthocyanin, delphinidin-3-O-glucoside (DP-3-G), cyanidin-3-O-glucoside (CG-3-G), petunidin-3-O-glucoside (PT-3-G), peonidin-3-O-glucoside (PN-3-G) and malvidin-3-O-glucoside (MV-3-G) were measured by an High-performance liquid chromatography (HPLC) system (LC-100; Wufeng series, Shanghai, China) equipped with LC-P100 pump and operated by LC-WS100 software, according to Simoes et al. (2009) with modification. Skin extracts were filtrated by a membrane filter (0.45 μm, MillexHV; Millipore, Bedford, MA, USA) and a volume of 20 μL of solution was injected directly into the HPLC system. The samples were analyzed using a BRISA LC2 C18 column (5 μm particle size, 250 × 4.6 mm I.D.). The gradients profile consisted of two eluents: (A) acetonitrile/water/formic acid at a ratio of 50:45:5 (v/v/v) and (B) water/formic acid at a ratio of 95:5 (v/v). The gradients used were as follows: 0–3% A at 1 min, 3–15% A at 11 min, 15–25% A at 12 min, 25–30% A at 4 min, 30% A at 7 min, and constant for 5 min before the mobile phase returned to the initial conditions. The flow rate was 1.0 mL/min at 30 °C and detection were at 520 nm. Peaks were identified according to each peak of UV-Vis spectra corresponding to the spectra of standard and comparing with their retention times. Anthocyanin content was quantified using peak areas of external standards. Standard curves were constructed using peak area vs. concentration (linear calibration curves). The resultant individual anthocyanin was expressed as milligram per killogram berry FW.

### Statistical analysis

Statistical analyses were done by SPSS v19.0 (SPSS Inc., Chicago, IL, USA). Data were subjected to one-way analysis of variance. When there was a significant difference (*p* < 0.05), the mean separation was performed using Tukey’s significance test set at *p* < 0.05. Correlations between variables were performed by Pearson’s correlation coefficients in SPSS.

## Results

### Soil moisture at different sites of soil during winter and summer seasons

Significant differences in the soil moisture at three different sites of soil were observed during both winter and summer seasons (Table 2). In case of top soil (0–20 cm depth), the highest soil moisture (30.78% and 41.62%) in winter and summer seasons were recorded from the lower site which was significantly higher than the other two sites. The lowest soil moisture was recorded from the upper site (22.33% and 33.02%) in both growing seasons. Available soil moisture recorded from 20 to 40 cm depth also followed the similar trends like top soil, where the lower site had the highest soil moisture (42.32% and 55.81%) which was significantly higher than the middle (34.27% and 49.33%) and upper site (27.89% and 45.10%). Significant difference in soil moisture also found between winter and summer cropping cycle. Soil moisture was higher in the summer season compared to the winter season as expected.

**Table 2:** Available soil moisture level at three different sites of soil during summer and winter cropping cycle.

	Soil moisture % (0–20 cm depth)	Soil moisture % (20–40 cm depth)
**Sites**	**Winter**	**Winter**
Lower	30.78 ± 0.32^a^	42.32 ± 1.24^a^
Middle	27.27 ± 0.83^b^	34.27 ± 1.06^b^
Upper	22.33 ± 0.73^c^	27.89 ± 0.99^c^
Sig	*	*
	**Summer**	**Summer**
Lower	41.62 ± 1.05^a^	55.81 ± 1.12^a^
Middle	36.45 ± 0.48^b^	49.33 ± 0.36^b^
Upper	33.02 ± 0.54^c^	45.10 ± 0.88^c^
Sig	*	*
**Season**		
Winter	26.80 ± 1.3^b^	34.83 ± 1.61^b^
Summer	37.03 ± 1.27^a^	50.08 ± 2.16^a^
Sig	*	*

**Notes:**

Mean (±SE) with different letters are significantly different within sites and season (mean separation by Tukey’s HSD test at *p* < 0.05).

* Indicates significance at *p* = 0.05.

### Effects of different sites of soil on chlorophyll a fluorescence

**Figure 2:**
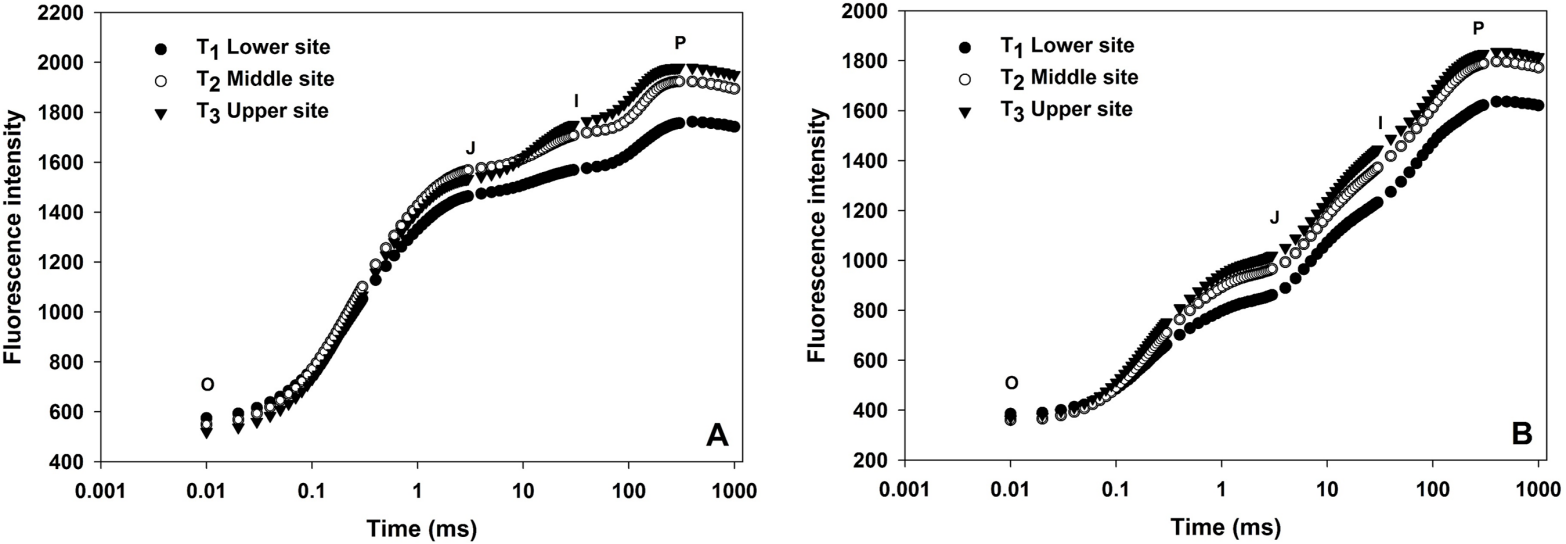
Effect of different sites of soil on the fast fluorescence induction curve (log time scale) of dark-adapted leaves during winter (A) and summer (B) cropping cycle.

OJIP transients from leaves of grapevine from three different sites shown in Fig. 2. All OJIP transients of leaves from three sites resulted in a typical polyphasic rise with basic steps of O-J-I-P in both winter and summer seasons. In the winter season, the fluorescence induction curve from the upper and middle site showed a considerable increase in J, I and P phases where there was low moisture compared to vines from lower sites (Fig. 2) indicating water stress due to the lower moisture content in the soil from upper and middle sites. A similar curve was also observed in the summer season although the overall fluorescence intensity value were lower than the winter season curve.

Chlorophyll a fluorescence provides several parameters about photosynthetic fluxes which are considered as a reliable indicator of plant stress monitoring. Table 3 and Fig. 2 show different technical parameters obtained from chlorophyll a fluorescent transient under three different soil moisture at the lower, middle and upper sites during winter and summer seasons. In this study, a significant gradual increase in minimal fluorescence (F_o_) was observed from lower site to upper site (592.94, 728.55 and 816.89, respectively) in the winter cropping cycle. Similar trend was also recorded for the summer season (368.18, 457.33 and 576.55). Significant differences were observed in case of F_v_/F_m_ value, in the winter season which were 0.80, 0.75 and 0.71 for the lower, middle and upper sites, respectively. Similarly, in the summer season, F_v_/F_m_ value gradually decreased from lower sites to upper sites (0.83, 0.81 and 0.75, respectively). When comparing the two seasons, there was significant difference between summer and winter in all the JIP parameters like F_o_, F_m_, F_v_ and F_v_/F_m_.

**Table 3:** Effects of vineyard sites of soil on Chl-a fluorescence kinetics during winter and summer cropping cycle.

	F_o_	F_m_	F_v_	F_v_/F_m_
**Sites**	**Winter**	**Winter**	**Winter**	**Winter**
Lower	592.94 ± 4.37^c^	3058.50 ± 26.43^a^	2465.60 ± 26.65^a^	0.80 ± 0.003^a^
Middle	728.55 ± 15.47^b^	2911.60 ± 7.91^b^	2183.00 ± 22.39^b^	0.75 ± 0.006^b^
Upper	816.89 ± 9.69^a^	2832.90 ± 9.62^c^	2016.00 ± 17.17^c^	0.71 ± 0.003^c^
Sig	*	*	*	*
	**Summer**	**Summer**	**Summer**	**Summer**
Lower	368.18 ± 17.19^c^	2083.90 ± 11.26^a^	1750.60 ± 18.12^a^	0.83 ± 0.003^a^
Middle	457.33 ± 28.94^b^	1820.50 ± 35.97^b^	1486.90 ± 40.25^b^	0.81 ± 0.01^a^
Upper	576.55 ± 8.06^a^	1641.80 ± 35.09^c^	1230.00 ± 11.97^c^	0.75 ± 0.02^b^
Sig	*	*	*	*
**Season**				
Winter	712.79 ± 33.01^a^	2934.30 ± 34.11^a^	2221.50 ± 66.54^a^	0.75 ± 0.02^b^
Summer	467.36 ± 31.79^b^	1848.80 ± 65.90^b^	1489.10 ± 76.29^b^	0.80 ± 0.01^a^
Sig	*	*	*	*

**Notes:**

Mean (±SE) with different letters are significantly different within sites and season (mean separation by Tukey’s HSD test at *p* < 0.05).

**F**_o_, minimum fluorescence; **F**_m_, maximum fluorescence; **F**_v_, variable fluorescence; **F**_v_/**F**_m_, maximum quantum efficiency of photosystem II.

* Indicates significance at *p* = 0.05.

### Effects of different sites of soil on yield and berry physicochemical characteristics

Analysis of the data indicated that during the winter season, grapevine from the middle site provided the highest value regarding cluster per tree, cluster weight, thereby yielding per vine (8.87 kg), which was statistically higher than upper and lower sites. The lowest yield per vine (5.45 kg) was recorded from the lower site (Table 4). Data from the summer season also followed the similar trend with higher values in terms of the above same three parameters (Table 4). Significant difference was observed between winter and summer seasons regarding cluster weight and yield per vine but not the cluster per tree. Moreover, during both growing seasons, there was no significant difference among the sites of soil regarding berry width, length and individual berry weight although data from the summer season was significantly higher than winter.

**Table 4:** Effect of vineyard sites of soil on yield and berry physicochemical properties during winter and summer cropping cycle.

	Cluster wt. (g)	Cluster/tree	Yield (kg)	Ind. fruit wt. (g)	Width (mm)	Length (mm)	TSS (°Brix)	pH	Acidity (%)	TSS/acidity
**Sites**	**Winter**	**Winter**	**Winter**	**Winter**	**Winter**	**Winter**	**Winter**	**Winter**	**Winter**	**Winter**
Lower	455.23 ± 11.77^b^	12.00 ± 0.58^b^	5.45 ± 0.14^c^	5.47 ± 0.22	20.59 ± 0.16	23.23 ± 0.21	17.17 ± 0.17^b^	3.53 ± 0.03	0.50 ± 0.008^a^	34.11 ± 0.30^b^
Middle	511.85 ± 3.53^a^	17.33 ± 0.33^a^	8.87 ± 0.20^a^	5.22 ± 0.08	20.16 ± 0.24	22.18 ± 0.24	18.33 ± 0.17^a^	3.60 ± 0.06	0.46 ± 0.003^b^	39.57 ± 0.33^a^
Upper	473.37 ± 4.92^b^	14.33 ± 0.88^b^	6.78 ± 0.38^b^	5.12 ± 0.02	20.06 ± 0.07	22.12 ± 0.40	19.00 ± 0.29^a^	3.87 ± 0.03	0.45 ± 0.008^b^	41.95 ± 1.29^a^
Sig	*	*	*	ns	ns	ns	*	ns	*	*
	**Summer**	**Summer**	**Summer**	**Summer**	**Summer**	**Summer**	**Summer**	**Summer**	**Summer**	**Summer**
Lower	503.87 ± 17.71^b^	11.67 ± 0.88^b^	5.86 ± 0.39^c^	6.80 ± 0.18	21.64 ± 0.25	23.72 ± 0.14	16.90 ± 0.05^b^	3.36 ± 0.03	0.69 ± 0.06^a^	24.63 ± 2.14^b^
Middle	615.08 ± 14.41^a^	18.67 ± 0.88^a^	11.49 ± 0.64^a^	6.43 ± 0.13	21.20 ± 0.24	23.13 ± 0.36	17.50 ± 0.25^b^	3.43 ± 0.07	0.61 ± 0.04^ab^	28.78 ± 1.67^b^
Upper	593.86 ± 22.24^a^	15.00 ± 0.58^b^	8.92 ± 0.57^b^	6.15 ± 0.16	20.65 ± 0.29	22.81 ± 0.23	18.33 ± 0.17^a^	3.65 ± 0.08	0.50 ± 0.01^a^	36.49 ± 1.26^a^
Sig	*	*	*	ns	ns	ns	*	ns	*	*
**Season**										
Winter	480.15 ± 9.18^b^	14.56 ± 0.84^a^	7.04 ± 0.51^b^	5.27 ± 0.09^b^	20.27 ± 0.12^b^	22.62 ± 0.29^b^	18.17 ± 0.29^a^	3.66 ± 0.06^a^	0.47 ± 0.01^b^	38.55 ± 1.22^a^
Summer	570.93 ± 19.37^a^	15.11 ± 1.09^a^	8.76 ± 0.86^a^	6.46 ± 0.13^a^	21.17 ± 0.19^a^	23.23 ± 0.19^a^	17.58 ± 0.23^b^	3.48 ± 0.05^b^	0.60 ± 0.04^a^	29.97 ± 1.94^b^
Sig	*	ns	*	*	*	*	*	*	*	*

**Notes:**

Mean (±SE) with different letters are significantly different within sites and season (mean separation by Tukey’s HSD test at *p* < 0.05).

Asterisks (*) and ns indicate significance at *p* = 0.05 and non-significant, respectively.

As shown in Table 4, the highest TSS (°Brix) value during both growing seasons was obtained from the upper site which was statistically similar to the middle site. While in both seasons, the lowest TSS value was recorded from the lower site. Overall, TSS value was higher in the winter season compared to the summer season grape. There was no significant difference in pH value in different sites; however, pH was higher in winter grapes.

### Effects of different sites of soil on phenolics, flavonoid and antioxidant properties

Table 5 shows the differences those were observed in determination of total phenolics, flavonoid and antioxidant properties of table grape as influenced by the soil levels. Comparatively, significantly higher contents of total phenolics, flavonoid and antioxidant properties were recorded from the middle site of the vineyard compared to upper and lower sites during both the seasons. Comparing both growing seasons, significantly higher values were recorded from winter for all three parameters (Table 5).

**Table 5:** Effect of vineyard sites of soil on total phenolics, flavonoid, and antioxidant properties during winter and summer cropping cycle.

	Total phenolics (mg/100 gFW)	Flavonoid (mg/100 gFW)	Antioxidant (mMTE/gFW)
**Sites**	**Winter**	**Winter**	**Winter**
Lower	499.80 ± 15.94^b^	1295.00 ± 42.62^b^	9.92 ± 0.55^b^
Middle	652.13 ± 11.93^a^	1888.00 ± 111.07^a^	14.45 ± 0.31^a^
Upper	605.93 ± 33.54^a^	1660.70 ± 75.32^a^	12.60 ± 0.59^a^
Sig	*	*	*
	**Summer**	**Summer**	**Summer**
Lower	431.27 ± 14.59^c^	1131.30 ± 14.59^c^	9.06 ± 0.44^b^
Middle	607.27 ± 13.81^a^	1636.00 ± 23.96^a^	13.03 ± 0.72^a^
Upper	513.60 ± 14.84^b^	1521.90 ± 14.32^b^	11.88 ± 0.35^a^
Sig	*	*	*
**Season**			
Winter	585.96 ± 25.07^a^	1614.60 ± 95.45^a^	12.32 ± 0.70^a^
Summer	517.38 ± 26.14^b^	1429.70 ± 76.94^b^	11.33 ± 0.64^b^
Sig	*	*	*

**Notes:**

Mean (±SE) with different letters are significantly different within sites and season (mean separation by Tukey’s HSD test at *p* < 0.05).

* Indicates significance at *p* = 0.05.

### Effects of different sites of soil on individual and total anthocyanin

**Figure 3:**
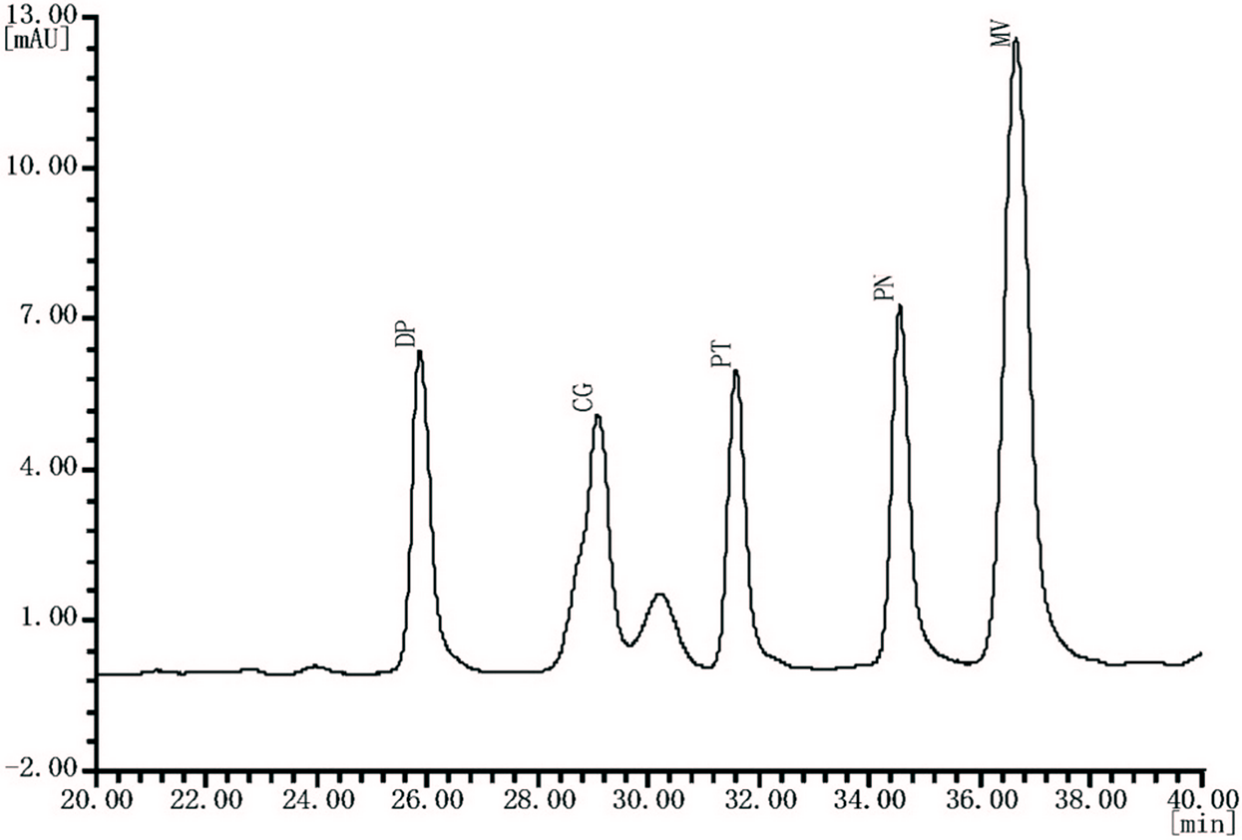
Typical HPLC chromatogram of anthocyanin extracts recorded at 520 nm of “Summer Black” table grape. DP, delphinidin-3-O-glucoside; CG, cyanidin-3-O-glucoside; PT, petunidin-3-O-glucoside; PN, peonidin-3-O-glucoside; MV, malvidin-3-O-glucoside.

The chromatograms of table grape obtained from skin extract eluted in the following order: DP-3-G, CG-3-G, PT-3-G, PN-3-G and MV-3-G (Fig. 3). The middle site provided the highest values regarding DP-3-G, CG-3-G, PT-3-G, PN-3-G and MV-3-G contents in our study during both winter and summer seasons (Table 6). Among the five studied individual anthocyanin MV-3-G was obtained in a higher amount than the rest of the anthocyanin in both the seasons. Total anthocyanin content varied between 791.20–893.89 mg kg^−1^ for this variety in winter and 587.6–771.71 mg kg^−1^ in the summer season. The middle site provided the highest total anthocyanin content during both growing seasons (Table 6). However, grapes from the winter season had a higher total and individual anthocyanin compared to the summer season.

**Table 6:** Effect of vineyard sites of soil on individual and total anthocyanin content during winter and summer cropping cycle.

	DP-3-G (mg/kg)	CG-3-G (mg/kg)	PT-3-G (mg/kg)	PN-3-G (mg/kg)	MV-3-G (mg/kg)	Total anthocyanin (mg/kg)
**Sites**	**Winter**	**Winter**	**Winter**	**Winter**	**Winter**	**Winter**
Lower	186.90 ± 1.38^b^	40.03 ± 0.83^b^	131.91 ± 2.38^b^	94.20 ± 3.90	250.35 ± 11.75^b^	791.20 ± 17.44^b^
Middle	208.98 ± 3.70^a^	50.76 ± 1.71^a^	150.41 ± 4.79^a^	97.57 ± 3.34	318.37 ± 4.53^a^	893.89 ± 11.43^a^
Upper	193.50 ± 1.99^b^	46.78 ± 0.89^a^	148.83 ± 2.34^a^	96.78 ± 1.89	313.56 ± 5.21^a^	828.34 ± 14.61^b^
Sig	*	*	*	ns	*	*
	**Summer**	**Summer**	**Summer**	**Summer**	**Summer**	**Summer**
Lower	115.64 ± 2.94^b^	22.02 ± 2.19^b^	114.53 ± 2.75^b^	61.83 ± 8.90^b^	205.41 ± 3.12^b^	587.06 ± 14.05^b^
Middle	182.80 ± 8.72^a^	36.89 ± 2.03^a^	140.55 ± 6.32^a^	95.47 ± 3.81^a^	298.65 ± 9.13^a^	771.71 ± 36.39^a^
Upper	148.34 ± 14.57^ab^	34.38 ± 2.89^a^	135.01 ± 5.63^ab^	81.62 ± 5.10^ab^	261.97 ± 11.51^a^	680.38 ± 7.78^ab^
Sig	*	*	*	*	*	*
**Season**						
Winter	196.46 ± 3.51^a^	45.85 ± 1.67^a^	143.72 ± 3.40^a^	96.18 ± 1.65^a^	294.09 ± 11.64^a^	837.81 ± 16.71^a^
Summer	148.93 ± 10.89^b^	31.09 ± 2.59^b^	130.03 ± 4.72^b^	79.64 ± 5.82^b^	255.35 ± 14.23^b^	679.72 ± 29.02^b^
Sig	*	*	*	*	*	*

**Notes:**

Mean (±SE) with different letters are significantly different within sites and season (mean separation by Tukey’s HSD test at *p* < 0.05).

DP-3-G, delphinidin-3-O-glucoside; CG-3-G, cyanidin-3-O-glucoside; PT-3-G, petunidin-3-O-glucoside; PN-3-G, peonidin-3-O-glucoside; MV-3-G, malvidin-3-O-glucoside.

Asterisks (*) and ns indicates significance at *p* = 0.05 and non-significant, respectively.

### Correlation between different parameters

Pearson’s correlation coefficient was computed to identify the relationship between different variables. As shown in Tables 7 and 8, significant positive correlations were found between TSS and TSS/acidity (*r* = 0.959, *p* = 0.01; *r* = 796, *p* = 0.05, respectively), also TSS and pH (*r* = 0.783, *p* = 0.05; *r* = 0.915, *p* = 0.01, respectively) during winter and summer seasons; while significant negative correlation was observed between TSS and acidity (*r* = −0.821, *p* = 0.01; *r* = −0.626,) in both growing seasons. Yield per vine was positively correlated with antioxidant and total anthocyanin in the winter season, and phenolics, flavonoid, antioxidant and total anthocyanin in the summer season. A positive correlation was found between soil moisture and acidity, individual fruit weight, berry width in both the seasons. However, negative correlation was observed between soil moisture and TSS, TSS/acidity, phenolics and flavanoid, antioxidant and total anthocyanin during winter and summer cropping cycle.

**Table 7:** Pearson’s correlation coefficient between different parameters, recorded from the “Summer Black” table grape during the winter season.

Parameters	1	2	3	4	5	6	7	8	9	10	11	12	13	14	15	16
1	1	0.875**	−0.276	−0.181	−0.246	0.551	0.649	0.673*	−0.842**	−0.597	0.865**	−0.899**	0.602	−0.526	−0.471	−0.276
2		1	−0.489	−0.279	−0.441	0.485	0.608	0.715*	−0.902**	−0.798**	0.790*	−0.881**	0.574	−0.537	−0.604	−0.344
3			1	0.744*	0.984**	−0.328	−0.307	−0.550	0.557	0.223	−0.600	0.583	0.322	0.702*	0.846**	0.805**
4				1	0.851**	−0.098	−0.355	−0.480	0.353	−0.032	−0.401	0.375	0.322	0.846**	0.723*	0.807**
5					1	−0.278	−0.329	−0.557	0.514	0.147	−0.568	0.542	0.359	0.772*	0.856**	0.847**
6						1	0.851**	0.663	−0.303	−0.157	0.687*	−0.496	−0.107	−0.473	−0.320	−0.064
7							1	0.832**	−0.412	−0.196	0.730*	−0.577	0.026	−0.685*	−0.505	−0.227
8								1	−0.564	−0.280	0.829**	−0.711*	0.014	−0.700*	−0.726*	−0.406
9									1	0.783*	−0.821**	0.959**	−0.529	0.489	0.656	0.491
10										1	−0.430	0.642	−0.723*	0.027	0.193	−0.043
11											1	−0.948**	0.168	−0.664	−0.738*	−0.509
12												1	−0.389	0.581	0.715*	0.513
13													1	0.140	0.151	0.246
14														1	0.801**	0.798*
15															1	0.886**
16																1

**Notes:**

1, soil moisture (top soil); 2, soil moisture (20–40 cm depth); 3, cluster/tree; 4, cluster weight (g); 5, yield (kg vine^−1^); 6, individual berry weight (g); 7, berry width (mm); 8, berry length (mm); 9, TSS (°Brix); 10, pH; 11, acidity (%); 12, TSS/acidity; 13, total phenolics (mg 100 g^−1^ FW); 14, flavonoid (mg 100 g^−1^ FW); 15, antioxidant (mMTE g^−1^); 16, total anthocyanin (mg kg^−1^).

* Significant correlation (*p* ≤ 0.05).

** Significant correlation (*p* ≤ 0.01).

**Table 8:** Pearson’s correlation coefficient between different parameters, recorded from the “Summer Black” table grape during the summer season.

Parameters	1	2	3	4	5	6	7	8	9	10	11	12	13	14	15	16
1	1	0.919**	−0.482	−0.617	−0.529	0.787*	0.765*	0.630	−0.797*	−0.641	0.875**	−0.923**	−0.535	−0.783*	−0.698*	−0.493
2		1	−0.568	−0.638	−0.595	0.683*	0.619	0.665	−0.839**	−0.690*	0.692*	−0.816**	−0.492	−0.765*	−0.627	−0.542
3			1	0.761*	0.978**	−0.278	−0.133	−0.299	0.300	0.091	−0.267	0.260	0.844**	0.869**	0.712*	0.927**
4				1	0.876**	−0.469	−0.472	−0.698*	0.673*	0.471	−0.359	0.441	0.854**	0.883**	0.789*	0.874**
5					1	−0.335	−0.230	−0.433	0.415	0.201	−0.291	0.306	0.894**	0.910**	0.768*	0.965**
6						1	0.968**	0.731*	−0.776*	−0.780*	0.838**	−0.871**	−0.446	−0.594	−0.743*	−0.236
7							1	0.766*	−0.797*	−0.799**	0.811**	−0.858**	−0.399	−0.530	−0.692*	−0.164
8								1	−0.891**	−0.891**	0.395	−0.557	−0.513	−0.572	−0.690*	−0.427
9									1	0.915**	−0.626	0.796*	0.373	0.591	0.605	0.353
10										1	−0.532	0.682*	0.191	0.366	0.440	0.171
11											1	−0.958**	−0.320	−0.571	−0.555	−0.205
12												1	0.312	0.597	0.584	0.210
13													1	0.923**	0.898**	0.898**
14														1	0.900**	0.876**
15															1	0.697*
16																1

**Notes:**

1, soil moisture (top soil); 2, soil moisture (20–40 cm depth); 3, cluster/tree; 4, cluster weight (g); 5, yield (kg vine^−1^); 6, individual berry weight (g); 7, berry width (mm); 8, berry length (mm); 9, TSS (°Brix); 10, pH; 11, acidity (%); 12, TSS/acidity; 13, total phenolics (mg 100 g^−1^ FW); 14, flavonoid (mg 100 g^−1^ FW); 15, antioxidant (mMTE g^−1^); 16, total anthocyanin (mg kg^−1^).

* Significant correlation (*p* ≤ 0.05).

** Significant correlation (*p* ≤ 0.01).

## Discussion

The present study was conducted to investigate the effect of within-vineyard soil variability on chlorophyll fluorescence, yield and quality parameters of table grape grown under double cropping viticulture system in the protected condition as influenced by soil moisture. The vineyard was divided vertically into three sites: upper, middle and lower. The data on soil moisture content indicate that there was significant difference among the treatments in soil moisture on top soil as well as soil from 20–40 cm depth in both winter and summer seasons. As expected, significantly higher soil moisture was recorded from the summer season compared to the winter season. The experimental vineyard being a hill slope, although upper canopy was covered by polythene film, the lower site had a chance of getting moisture from nearby low land as well as rainfall water as surface runoff and might have been the reason for high moisture content in the bottom site compared to middle and upper sites.

In our study, the OJIP transients data showed a polyphasic rise in the basic steps of O-J-I-P in both the seasons.

OJIP transients generally shows reduction of electron transport in PSII. During winter and summer seasons, the upper and middle site showed more detectable increase in the J, I and P phases compared to the lower site could be derived from the significant reduction of moisture in the upper and the middle sites. Similar results were obtained by Ghotbi-Ravandi et al. (2016) where moderate water loss (50% water holding capacity, WHC) did not show any considerable change with control condition (70% WHC), however, 30% WHC and 10% WHC showed significant difference in O-J-I-P steps. Higher fluorescent intensity was recorded in the winter season compared to the summer season in terms of lower, middle and upper sites which indicates more water stress in the grapevine in the winter season. This might be explained by the fact that in the summer season natural rainfall is higher which increased soil moisture to reduce the fluorescent intensity in JIP test (Ghotbi-Ravandi et al., 2016).

In the case of stress physiology studies F_o_, F_m_, F_v_, and F_v_/F_m_ values are the widely used chlorophyll fluorescence parameters (Fu, Li & Wu, 2012; Murchie & Lawson, 2013). F_o_ indicates the minimal fluorescence intensity levels when all the RCs related with the photosystem is assumed to be open (dark adapted) (Gorbe & Calatayud, 2012). An increase in F_o_ represents degradation in PSII or interference in the transfer of energy into the RC (Calatayud, Roca & Martínez, 2006). Generally, it is observed that under full stress condition, F_o_ is increased, while F_v_/F_m_ ratio is reduced (Maxwell & Johnson, 2000). In the current study, in the winter season, F_o_ significantly increased in upper site which might be due to low humidity status in the upper zone soil. Interestingly, in the summer season F_o_ value in all the lower, middle and upper sites were significantly lower than the value obtained in the winter season which clearly indicates the reduction of water stress in grapevine compared to winter. These results are in agreement with other researchers (Calatayud, Roca & Martínez, 2006; Fu, Li & Wu, 2012). A significant difference from F_m_, F_v_ and F_v_/F_m_ values were obtained. F_o_ value significantly decreased with increasing moisture whereas, F_m_, F_v_ and F_v_/F_m_ values were significantly increased with increasing moisture. The maximum quantum yield of primary photochemistry (F_v_/F_m_) which is a reliable indicator of the physiological state of the plant against environmental stress (Mehta, Allakhverdiev & Jajoo, 2010) was significantly decreased with decreasing moisture content in the soil. Under controlled conditions, F_v_/F_m_ value is near 0.8–0.83 for most of the plants (Björkman & Demmig, 1987; Ghotbi-Ravandi et al., 2016) which is equivalent to the photosynthetic rate of plants. In our study, in the winter season, F_v_/F_m_ value for the lower, middle and upper sites was 0.80, 0.75 and 0.71, respectively. This results seems to indicate that plants from the lower sites received optimum moisture from the soil, whereas plants from the middle sites had moderate stress and plants from upper sites received low moisture from soil. However, in the summer season, F_v_/F_m_ value was recorded as 0.83, 0.81 and 0.75, respectively. This can explain the reason for the increased grape yield from the middle sites as because the value is withing the optimum range of 0.80–0.83. In a previous study, Zulini et al. (2007) reported that at severe level of drought stress in grapevine, F_v_/F_m_ value decreased below 0.70 which corroborates with the value recorded in our study. As significant difference was observed among three different sites, in terms of chlorophyll fluorescence as well as soil moisture, it is clear that JIP test is a reliable indicator to determine the water stress condition in grapevine. The reason behind the lowest yield obtained from upper sites during both winter and summer seasons can be explained with significant lower moisture content thereby lower F_v_/F_m_ value. Middle layer resulted in higher yield compared to other two sites, indicating that, albeit F_v_/F_m_ data was significantly lower than the bottom site but the yield was not reduced. This could be explained by the fact that sustained deficit irrigation and regulated deficit irrigation has been found to save irrigation water without effecting the yield (Fereres & Evans, 2006). In this study moderate stress due to soil moisture did not reduce the yield as reported in other studies (Fereres & Evans, 2006).

As of chlorophyll fluorescence, significantly higher F_v_/F_m_ ratio was recorded from lower site, in our study the lower site resulted in the lowest yield, the reason might be due to the lower site was a valley area and there were chances to get moisture from other sources due to low land. Moreover, throughout the experiment visually the lower site appeared to look wet which have caused an increase in plant vegetative growth and less fruit production as low number of cluster per trees were recorded in the lower site with lower cluster weight in comparison to the middle and upper sites. According to chlorophyll fluorescence data lowest moisture content in the upper site induced reduction of maximum quantum yield of PSII which might be the reason due to lower cluster number and cluster weight with lower berry length, diameter and individual berry weight from the upper sites. The highest yield of grape was obtained from the middle sites. This could be due to lower vegetative growth and proper assimilation of food in reduced irrigation as no irrigation was applied after veraison. Deficit irrigation has been successfully applied for the yield improvement of peaches, apple and other horticultural fruits although few on table grape (Blanco, Faci & Negueroles, 2010). It has been reported that irrigation above 80% of the Evapotranspiration (ETc) did not increase the yield of grape (Williams, Grimes & Phene, 2010). Fereres & Evans (2006) also concluded that full ETc for the tree crops and vines are not always necessary in many cases. TSS and acidity are two important parameters which improve fruit quality (Chanana & Gill, 2008). Increased TSS with decreased firmness and acidity under deficit irrigation were reported previously (Elansary et al., 2005) in case of “Muscat of Alexandria” variety.

As of total phenolics, flavonoid and antioxidant capacity, plants from middle site in both winter and summer growing season resulted in higher quality data. These findings agreed with the values reported by Koundouras et al. (1999) who found higher concentrations of phenolics in berries from vines grown in soils with a certain water deficit during the ripening period. Matthews & Anderson (1988) also reported that both early and late season water deficits increased juice and skin phenolics in berries of Cabernet Franc vines. In our study significantly higher total phenolic, flavonoid and antioxidant capacity were recorded from winter berries compared to summer berries. In other double cropping viticulture system studies, also reported similar higher value from the winter season (Favero et al., 2011; Xu et al., 2011).

For table grape, fruit color is an important quality attribute. The grape berry contains anthocyanin in the skins which is the most prominent pigment for the coloration of the grape skin (Romero et al., 2008). The total anthocyanin as well as individual anthocyanin was higher in the middle sites compared to upper and lower sites in both the growing seasons. Our findings were similar to those of Wang et al. (2013) and Xi et al. (2016) with the total anthocyanin values (800 mg kg^−1^) identified from the same table grape variety. Among the five studied individual anthocyanin MV-3-G was obtained in a higher amount than the rest of the anthocyanin in both season grapes. Previous studies have also highlighted that a water deficit period shortly after veraison, when most anthocyanin synthesis occurs, resulted in higher levels of skin anthocyanin at harvest (Santesteban, Miranda & Royo, 2011). Water deficits were shown to consistently promote higher concentrations of anthocyanin in red wine grapes (Pascual, Joseignacio & Adrian, 2010; Terry & Kurtural, 2011). In our study, we also did not supply irrigation water after veraison to induce anthocyanin accumulation compared to vines that had chance to receive more water than the vine on lower sites. Similarly, Koundouras et al. (2009) noted that “Cabernet Sauvignon” vine subjected to DI (50% of ETc), resulted in an increase in flavor at harvest and higher accumulation of anthocyanin in berries due to pre-veraison drought stress. Dudareva, Pichersky & Gershenzon (2004) noted that the production and accumulation of aroma volatiles in fruit could be influenced by soil moisture. Elansary et al. (2005) also demonstrated that deficit irrigation increased the aroma content in “Muscat of Alexandria” grapes. In our study, berries from the winter season accumulated more anthocyanin compared to summer berries. Similar results have been reported in several studies (Favero et al., 2011; Xu et al., 2011).

## Conclusions

In the present research, we investigated the effects of within-vineyard variability in soil on chlorophyll fluorescence, yield and quality parameters of table grape under double cropping system, popularly practices in Southern China in the protected condition as influenced by soil moisture. It was concluded that summer black table grapes grown at different height of a vineyard displayed a significant difference in grape yield and quality attributes in both winter and summer seasons. We focused on soil moisture as the major parameter associated with the different sites of soil in the vineyard and found that variation in soil moisture significantly affected grape yield and chemical composition in both growing seasons. As the soil type was similar in all three sites, any variation in the data obtained here could be explained due to the variation in the available soil moisture. In this current study, JIP-test parameters also changed under different moisture level. Moreover, PSII activity was also influenced by the different sites of soil due to changing soil moisture level. These results indicate that chlorophyll fluorescence OJIP transient can be used as a sensitive tool to determine moisture stress in a vineyard with variation in altitude in a small scale. A greater understanding of these aspects will be useful for the grape growers for better utilization of different parts of the vineyard with proper irrigation management to maintain uniform moisture required for the plant to get a higher yield with quality fruit attributes. Grape is mainly a summer crop, and harvesting in June–July causes significant loss due to excessive rainfall. However, this study shows that protected shelter of grapevines with minimum irrigation and the use of sensitive chlorophyll, a fluorescence OJIP test, can help to better utilize the soil variability and irrigation water to increase the quality attributes of table grapes. This study also highlights the potential of using a double cropping viticulture system to harvest two times in a year to maximize the production of quality grapes.

## Supplemental Information

10.7717/peerj.5592/supp-1 Supplemental Information 1 Raw data.

## Additional Information and Declarations

### Field Study Permissions

The following information was supplied relating to field study approvals (i.e., approving body and any reference numbers): Field experiments were approved by the authority of the Fujian Jiujiu Tianchen Eco-Agri Ltd. who owns the vineyard where the experiments were conducted.

### Data Availability

The following information was supplied regarding data availability: The raw data are provided in a Supplemental File.
